# Conservative Treatment of Unicuspid Aortic Valve with Newly Diagnosed Type A Aortic Dissection

**DOI:** 10.21470/1678-9741-2020-0061

**Published:** 2020

**Authors:** Vera Graup, Lukas Meier, Francesco Maisano, Ahmed Ouda

**Affiliations:** 1 Department of Cardiac Surgery, University Hospital Zürich, Zürich, Switzerland.; 2 Department of Cardiology, University Hospital Zürich, Zürich, Switzerland.

**Keywords:** Aortic Valve Disease 1, Aortic Valve, Aorta, Thoracic, Dilatation, Heart Valve Diseases, Aortic Diseases, Heart Defects, Congenital

## Abstract

We present a case of a 36-year-old male patient with known arthrogryposis multiplex congenita and an associated unicuspid aortic valve. The patient later developed a significant aneurysm of the ascending aorta, however refused surgical intervention and missed follow-up appointments for 5 years. During an urgent, general practitioner-initiated transthoracic echocardiography follow-up, a chronic type A aortic dissection was diagnosed as a result of progressive aortic dilatation. Due to the stationary pressure gradients and non-progressive leaflet fibrosis, a conservative approach for to the unicuspid aortic valve was chosen, combined with replacement of the ascending aorta and partial replacement of the aortic arch.

**Table:** 

Abbreviations, acronyms & symbols	
CT	= Computed tomography
TTE	= Transthoracic echocardiography
UAV	= Unicuspid aortic valve

## INTRODUCTION

Unicuspid aortic valve is a rare congenital cardiac anomaly with a known risk for progressive cusp degeneration and dilatation of the ascending aorta. Herein, we describe a successful conservative approach to a unicuspid aortic valve in a setting of a chronic type A aortic dissection.

## CASE REPORT

**Fig. 1:**
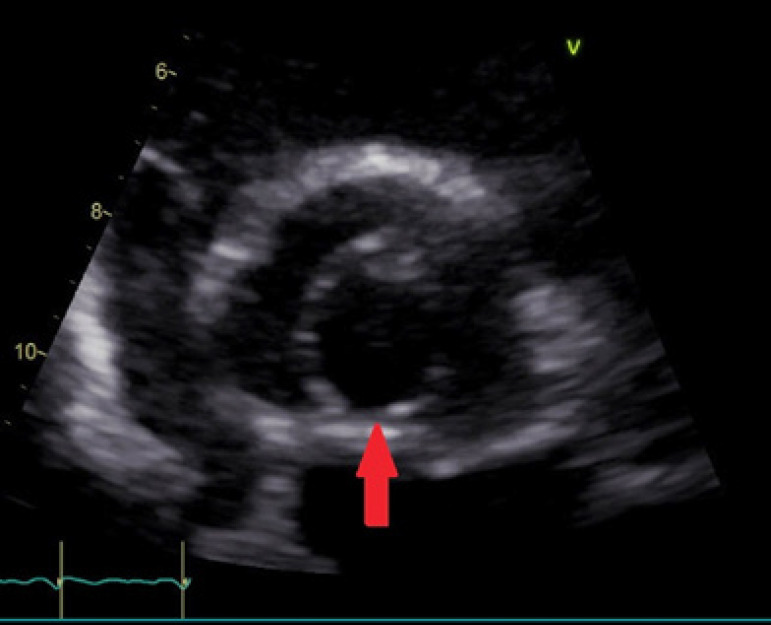
TTE image of UAV during a standard follow-up appointment. The red arrow indicates the aortic commissure.

**Fig. 2:**
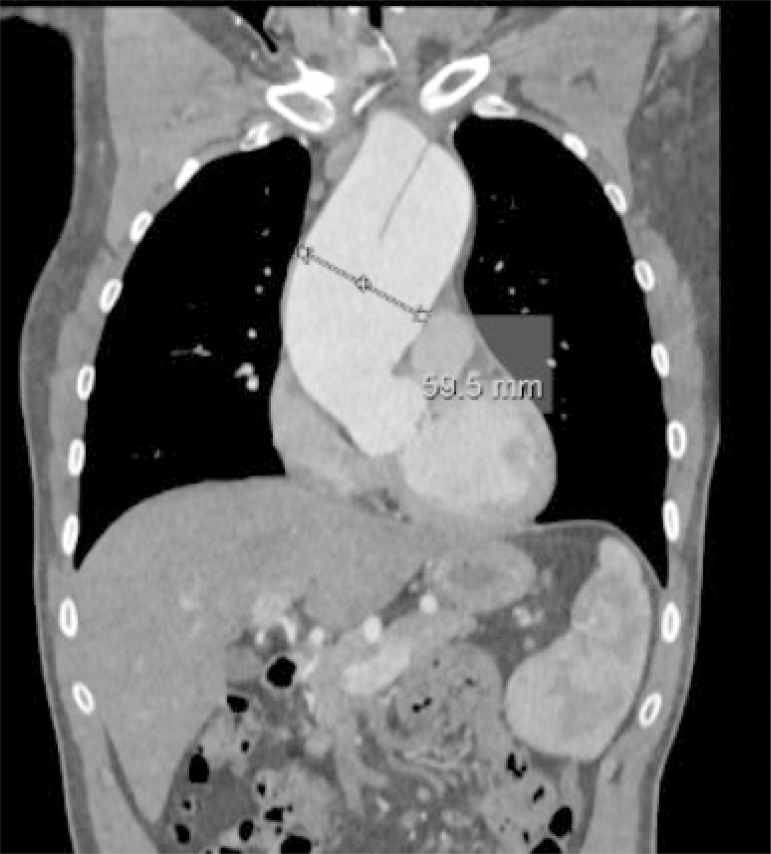
Thoraco-abdominal CT scan on the day of the first diagnosis, showing aortic aneurysm and dissection membrane.

We report a case of a 36-year-old man with known arthrogryposis multiplex congenita (Guerin-Stern syndrome) and a known unicuspid aortic valve (UAV). The patient was regularly followed up by transthoracic echocardiography (TTE), which excluded progression of aortic stenosis. In the ten years of TTE follow-up, the peak gradient ranged from 45 to 52 mmHg, with a mean gradient of 23 to 32 mmHg and the mean valve orifice area from 1.1 to 1.3 cm^2^ (representative TTE shown in Figure 1). At the age of 31, a 51-mm diameter aneurysm of the ascending aorta was diagnosed; however, the patient refused surgical treatment. After a 5-year window of patient non-compliance, an emergency TTE was initiated by the patient’s general practitioner, which showed stationary valve parameters (peak pressure of 38 mmHg, mean gradient of 23 mmHg, mean valve orifice area of 1.3 cm^2^) and a progression of the ascending aneurysm to 61 mm, and a chronic type A aortic dissection. The meticulous history-taking revealed an event of acute chest pain approximately 2 weeks prior to the examination, for which the patient did not seek treatment. A subsequently conducted computed tomography (CT) scan showed an aortic aneurysm with a type A dissection (representative CT scan shown in Figure 2) beginning from the sino-tubular junction to the proximal part of the descending aorta with continuation of the dissection into the brachiocephalic trunk.

**Fig. 3:**
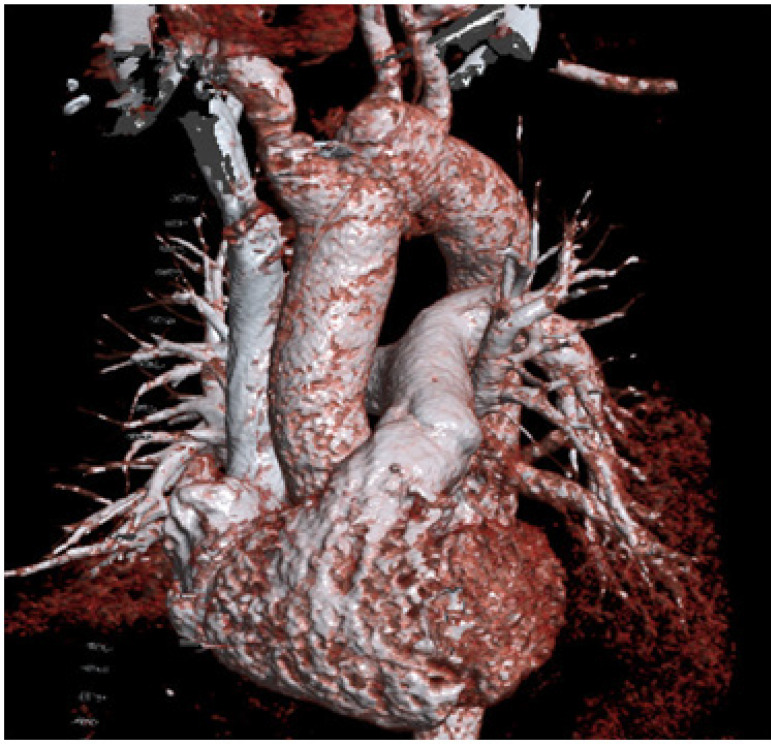
3D reconstruction of postoperative CT showing the replacement of the ascending aorta and the re-implanted brachiocephalic trunk.

Surgery was carried out under general anaesthesia using cardiopulmonary bypass and hypothermic circulatory arrest with selective antegrade cerebral perfusion. Careful inspection of the unicuspid aortic valve revealed slightly fibrosed valve leaflets. Considering that there had been ten years of non-progressive, mild stenosis of the aortic valve, the heart team decided not to replace the aortic valve with a mechanical prosthesis due to subsequent endocarditis and risk of stroke. Surgical treatment included the replacement of the supra-coronary aorta with a prosthesis (Gelweave Lupiae 28×40 mm; Vascutek Terumo Inc., Scotland, UK), as well as the replacement of the aortic arc with simultaneous reimplantation of the brachiocephalic trunk (Figure 3). Postoperatively, the patient spent one day on intensive care and was discharged on the 11^th^ postoperative day; medication consisting of aspirin 100 mg and bisoprolol 2.5 mg. Postoperative TTE showed the unicuspid aortic valve with a combined stenosis and insufficiency with a peak gradient of 23 mmHg, a mean pressure gradient of 12 mmHg and a mean orifice area of 1.8 cm^2^. Transthoracic echocardiographic follow-up after six months, one year, and two years after the intervention showed stationary parameters. The patient remains asymptomatic under unchanged medication at annual follow-up appointments 2 years after surgery.

## DISCUSSION

UAV is a rare congenital valve malformation characterized by a unique circular cusp with one commissure or, rarely, without commissure. It typically produces severe obstruction in infancy and is the most common malformation in children under one year of age with aortic stenosis. Rarely, it can also be seen in adults with an incidence of about around 0.02%^[1]^. It is known that BAV patients have an age-adjusted relative risk of 8.4 for developing aortic dissection when compared to patients with tricuspid aortic valve. However, the incidence of aortic dissection remains low (3.1 per 10,000 patients per year). Although UAV is far less frequent than BAV, the association with aortic dissections seems similar (7-12% in UAV and 4-6% in BAV)^[2]^.

Zhu et al.^[3]^ analysed the outcome of 149 adult UAV patients treated with aortic valve replacement/repair alone or combined with replacement of the ascending aorta. The mean maximum aortic diameter is 44±8 mm and involved the aortic root, ascending aorta and the aortic arch in various degrees. The authors found that combined aortic valve operation and aortic repair was associated with substantially better long-term survival than a valve operation alone. However, there are no data available in the literature regarding the outcome of UAV adult patients with type A aortic dissection treated with aortic repair and sparing of a well-functioning native UAV.

Here we report a case of UAV in association with a type A aortic dissection in which the surgical intervention was targeted solely to the ascending aorta, but did not include valve replacement.

UAV is known to be associated with other cardiac malformations, such as patent ductus arteriosus, coarctation of the aorta, and aortic aneurysms.

This common association of UAV and aortic aneurysms and hence aortic dissection is of interest to the treating cardiac surgeon. In the case presented here, initially the diagnosis of UAV was made in an otherwise cardio-pulmonary asymptomatic patient, hence no surgical intervention was indicated^[4]^. When the initial diagnosis of 51-mm diameter aortic aneurysm was made, the patient refused surgical intervention (indicated with any aortic aneurysm >50 mm in diameter)^[5]^. At the time of diagnosis of type A aortic dissection, an interdisciplinary heart team (cardiac surgeon, cardiologist and cardiac anaesthetist) decision had to be made about the surgical strategy, i.e. replacement of the supra-coronary aorta with/without simultaneous replacement of the aortic valve. In case of simultaneous valve replacement, a mechanical prosthesis would have to be chosen in such a young patient, resulting in long-term complication risks from oral anticoagulation. Considering the initial and to this point unchanged diagnosis of light aortic valve stenosis without insufficiency, the probability of the need for valve replacement in the next 10-15 years was estimated to be under 20%. Later, potentially minimally invasive intervention remained an option, depending on the long-term development of the patient. Hence, the decision was made to leave the natural valve *in situ*.

## CONCLUSION

This case report highlights the importance of interdisciplinary decisions by the heart team considering the immediate patient needs. These considerations changed the surgical technique in this case. To this date, the patient remains asymptomatic without oral anticoagulation.

**Table:** 

Authors' roles & responsibilities	
VG	Substantial contributions to the conception or design of the work; or the acquisition, analysis or interpretation of data for the work; final approval of the version to be published
LM	Substantial contributions to the conception or design of the work; or the acquisition, analysis or interpretation of data for the work; final approval of the version to be published
FM	Drafting the work or revising it critically for important intellectual content; final approval of the version to be published
AO	Substantial contributions to the conception or design of the work; or the acquisition, analysis or interpretation of data for the work; final approval of the version to be published

## References

[1] Mookadam F, Thota VR, Garcia-Lopez AM, Emani UR, Alharthi MS, Zamorano J (2010). Unicuspid aortic valve in adults: a systematic review. J Heart Valve Dis.

[2] Roberts CS, Roberts WC (1991). Dissection of the aorta associated with congenital malformation of the aortic valve. J Am Coll Cardiol.

[3] Zhu Y, Roselli EE, Idrees JJ, Wojnarski CM, Griffin B, Kalahasti V (2016). Outcomes after operations for unicuspid aortic valve with or without ascending repair in adults. Ann Thorac Surg.

[4] Baumgartner H, Falk V, Bax JJ, Bonis M, Hamm C, Holm PJ (2018). [2017 ESC/EACTS Guidelines for the management of valvular heart disease]. Kardiol Pol.

[5] Erbel R, Aboyans V, Boileau C, Bossone E, Di Bartolomeo R, Eggebrecht H (2014). [2014 ESC Guidelines on the diagnosis and treatment of aortic diseases]. Kardiol Pol.

